# Risk factors and distribution of oncogenic strains of human papilloma virus in women presenting for cervical cancer screening in Port Harcourt, Nigeria

**DOI:** 10.11604/pamj.2016.23.85.8510

**Published:** 2016-03-11

**Authors:** Nyengidiki Tamunomie Kennedy, Durugbo Ikechukwu, Bassey Goddy

**Affiliations:** 1 Department of Obstetrics and Gynaecology, University of Port Harcourt Teaching Hospital, Port Harcourt, Rivers State, Nigeria

**Keywords:** HPV, prevalence, risk factors, Port Harcourt, Nigeria

## Abstract

**Introduction:**

Human papilloma virus(HPV) accounts for most cases of cervical cancer with high risk HPV(hrHPV) genotypes largely responsible. The objective is to ascertain the distribution of oncogenic strains of human papilloma virus genotypes and predisposing risk factors in women presenting for cervical cancer screening in Nigeria.

**Methods:**

A cross-sectional study of 80 women who presented for cervical cancer screening. The biodata of the participants, the presence of risk factors to HPV were recorded and hrHPV were identified using PCR technique. The information obtained was processed using the SPSS version 20 software. Results were presented in tables, test of significance and association done using student's t-test and Odds ratio, with P value < 0.05 as significant.

**Results:**

The age range of patients was 19-62 years with prevalence of hrHPV of 10%. HrHPV are more in patients with more than one life time sexual partner (OR 1.26,95%CI 0.13-29.99), multiple sexual partners (OR 1.55, 95% CI 0.28-8.70), early coitarche (OR 1.57, 95% CI 0.14-15.00) and previous STI (OR 150, 95%CI 9.53-1979. 62). Four hrHPV genotypes: 16, 18, 31 and 35 were detected.

**Conclusion:**

HPV genotype 18 was predominant in Port Harcourt, Nigeria. High risk sexual behaviours are associated with acquisition of hrHPV.

## Introduction

Cervical cancer is a malignant lesion of the cervix. The squamous cell carcinoma constitute about 90-95% of cases while adenocarcinoma make up about 5-10% [1]. Worldwide, every two minutes, a woman dies of cervical cancer and it is the leading cause of cancer deaths with women in developing countries accounting for 85% of these deaths [2]. About 99% of cases of cervical cancer have been associated with human papilloma virus infection which is a sexually transmitted disease [2]. Out of about 200 types of HPV isolated so far, at least 30 different types target genital mucosa and approximately 15 of these are oncogenic [2, 3]. About 80% of women will acquire an HPV infection in their lifetime and HPV genotypes 16, 18, 45, 31 and 33 are the most common types associated with cervical cancer worldwide [2, 3]. About six percent of cervical cancer cases had been associated with multiple HPV infections [2]. HPV is a relatively small virus containing circular double-stranded deoxyribonucleic acid within a spherical shell (capsid). It infects cutaneous epithelium and mucosal epithelium (cervical and other anogenital mucosae). HPV is transmitted through sexual intercourse and/or genital skin-to-skin contact [3]. It is the most common sexually acquired infection in the world with a prevalence of about 50% in young sexually active adolescents [3, 4]. Most of the infections are self-limiting and harmless and the persistence of the oncogenic HPV types cause cervical cancer in women [4, 5]. The prevalence of HPV genotypes has been documented from different studies in different countries and regions [4–7]. However, the prevalence of HPV genotypes has not been well studied in the South -South region of Nigeria. It is based on this premise that this study seeks to determine the predominant high risk HPV genotypes in the region; in addition to elucidating the risk factors associated with this infection so as to facilitate measures of reducing the scourge of cervical cancer in the populace, which has been linked to human papilloma virus infection.

## Methods

This was a cross sectional prevalence study carried out, between August 2014 and December 2014 in the cancer screening centre of UPTH on women who presented for cervical cancer screening in the University of Port Harcourt Teaching Hospital, Port Harcourt. All women between the ages of 15 to 70 years were included in the study after due counseling and obtaining consent to be involved in the study. Women were excluded from the study if they refused to give consent, had total abdominal hysterectomy, had been treated for premalignant lesion of the cervix, were adequately immunized against the human papilloma virus or had received antineoplastic therapy or immunotherapy. The sample size was determined using the Leshe-kish formula for single proportion [8] which is n = Z^2^p(1-p)/d^2^; where P is the HPV prevalence in Ibadan Nigeria [4]. Allowing for 100% degree of accuracy and allowing a ten percent attrition, a total of 80 women who presented for cervical cancer screening were recruited. Patients were recruited as they presented until the required number was achieved.

A structured study proforma was administered on each of the women. The following information was obtained from the women: age, occupation level of education, marital status and parity. Other information obtained from the women included the history of smoking, age at coitarche; number of lifetime sexual partners and history of previous sexually transmissible infections. The presence of partners who had multiple sexual consorts was also ascertained. The phone numbers of the participants were collected for disclosure of the results, counseling and advice on further management where necessary.

The collection of the sample was done in the presence of a female nurse who also served as a chaperone. In collecting the sample, the women were placed in lithotomy position, the cervix was exposed using the bivalve speculum and then inspected. The specimen for the HPV genotyping was collected using the cyto-brush, cut short at the brush end and inserted into an already numbered specimen bottle, which contained a physiological saline. All collected samples with the cyto-brush were packaged in an ice pack in a container and were sent to the Safety Molecular Pathology laboratory at Enugu, Nigeria through a prearranged courier service. The Pathology department of the UPTH has collaboration with the Safety Molecular Pathology laboratory Enugu. The cervical samples were analyzed for the presence of all possible oncogenic strains of HPV DNA using Real time Polymerase Chain Reaction (PCR). In determining the HPV genotypes where present, a PCR mixture was prepared in a tube. The mixture contained the DNA sample (extracted from the cyto-brush scrapings), primers (forward and reverse primers) and reaction buffer from magnesium chloride. Other contents of the mixture included deoxynucleosides triphosphates (dNTPs) and Taq DNA polymerase.

Statistical analysis was done using computer software Statistical Package for Social Science version 20.0(SPSS Inc; Chicago USA). Frequency tables were generated and the results tested of significance using student t-test and chi-square. The age-specific prevalence and the contribution of other socio-demographic factors were computed. The risk of acquiring hrHPV was estimated with odds ratio. Statistical test of association was carried out at the level of significance set at P value <0.05 at 95% confidence interval. Ethical considerations: Ethical clearance for the study was obtained from the ethics committee of the Teaching Hospital before the commencement of the study.

## Results

A total of 80 women who met the inclusion criteria were recruited for the study. The age range of the participants was 19-62 years. The median age was 39 ± 5 years. Eight women out of the 80 women who participated in the study were positive for the oncogenic strains of human papilloma virus giving a prevalence of 10%.

In Table 1, the socio-demographic characteristics of the women showed that 50% (4) of the women who had HPV were less than 25 years. The peak age prevalence was between 30-39 years at 37%. Eighty seven percent of the women with HPV had 3 or more deliveries. Among women with hrHPV, 87.5%(7) had secondary or tertiary education (P = 0.08; OR 0.13, 95%CI 0.01-1.40). The distribution of HPV genotypes of the participants identified, showed that four hrHPV genotypes were detected in the eight women. The detected HPV types were 16, 18, 31 and 35. One woman had 2 different types of hrHPV (18 and 35). HPV type 18 accounted for 44% (4) of the detected HPV genotypes while type 16 and 35 accounted for 22.2% (2) each and human papilloma virus types 31 accounted for 11% (1).

**Table 1:** Socio-demographic characteristics of the participants

Variable	Frequency N = 80	hrHPV positive N = 8	Test of Significance
	F (%)	F (%)	
**Age (years)**			
<20	2(2.50)	1 (12.50)	
20-29	8(10.00)	3 (37.00)	
30-39	12 (15.00)	2 (25.00)	
40-49	28 (35.00)	1 (12.50)	
50-59	21(26.25)	1 (12.50)	
60-70	9 (11.25)	0(0.00)	
**Parity**			
0	5 (6.25)	0 (0.00)	P = 0.42 OR = 2.18 CI(0.25-18.68)
1-2	14 (17.50)	1 (12.50)	
3-4	26 (32.50)	2 (25.0)	
>5	35 (43.71)	5 (62.5)	
**Occupation**			
Housewife	12(15.00)	3 (37.50)	
Unskilled	49 (61.25)	4 (50.00)	
Skilled/professional	19 (23.75)	1 (12.50)	
**Educational Status**			
None	3 (37.50)	2 (25.00)	P = 0.08 OR = 0.13 Cl(0.01-1.40)
Primary	17 (21.25)	4 (50.00)	
Secondary	41 (51.25)	1 (12.50)	
Tertiary	19 (23.75)	1 (12.50)	

Table 2 discloses the risk factors identified in patients with oncogenic strains of HPV. Among the patients with oncogenic strains of HPV, 87.5% (7) had more than one lifetime sexual partner (OR 1.26 95%CI 0.13-29.99). Spouse with multiple sexual partners, age at early coitarche and previous history of sexually transmissible infection was associated with higher risk of acquisition of the high risk human papilloma virus (hrHPV) infection. The risk of smoking in patients, was also assessed and found not to be statistically significant and with no association to the presence of hrHPV (P 0.81;OR 0.0 95%CI.0.28-13-18). Use of hormonal and barrier contraceptive methods were not associated with the acquisition of oncogenic strains of the virus (See Table 2).

**Table 2:** Risk factors for human papilloma virus infection among patients with hrHPV

Variables	HPV negative	HPV positive	P value	OR (95%CL)
**Life time sexual** **Partners**				
0	0	0	0.67	1.26(0.13-29.99)
1	11	1		
>1	61	7		
**Spouse with multiple sexual partners**				
Yes	61	6	0.461	1.55(0.28-8.70)
No	11	2		
**Oral contraceptive** **Usage**				
Yes	19	1	0.35	0.40(0.02-3.65)
No	53	7		
Age at coitarche				
<15	6	1	0.54	1.57(0.14-15.00)
≥15	66	7		
**Smoking**				
Yes	2	0	0.81	0.00(0.00-41.24)
No	70	8		
Barrier contraceptive usage				
Yes	46	6	0.42	0.59(0.08-3.62)
No	26	2		
History of STI				
Yes	2	6	0.0000025	150(9.53-1979.62)
No	70	2		

hrHPV- high risk human papilloma virus

## Discussion

The prevalence of human papilloma virus (HPV) in this study was 10% which is similar to the adjusted global prevalence of 10.41% in a study on epidemiology and transmission dynamics of genital HPV infection [9]. However, higher prevalence of 24.8% and 21.6% were found in a population-based study in Ibadan and Okene, Nigeria respectively where oncogenic strains were identified in 19.7 and 16.6% respectively [4, 10]. Prevalence of 11% was also found in women who tested negative to human immunodeficiency virus in Lagos, Nigeria [11] though the retroviral status of patients was not evaluated in this survey. There is a global variation in the prevalence of oncogenic strains as evident by observations in Benin, West Africa and Puebla in Mexico where prevalence of 32.2% and 24.4% respectively had been noted [12, 13]. The study in Benin discovered that the high risk HPV genotypes of 16, 18, 35, 58 and 59 were found in 88% of the infections [12]. The HPV genotypes of 16, 18,31 and 35 found in this study was relatively similar to types 16,31,35 and 58 noticed in Ibadan, Nigeria [4]. Similarly, epidemiological studies in Ghana and South Africa revealed that types 16, 18, 35 and 45 were the most common HPV types in Sub-Saharan African women [14]. However, type 45 was not isolated in this study.

The human papilloma virus type 18 was the predominant genotype identified. A review of patients with proven cervical cancer in Ghana and South African also identified serotype 18 as being contributory in about 17.2% of cases reviewed [14]. The study in Mexico revealed that HPV type 18 contributed 35.7% of the detected HPV genotypes [13]. An interface of the various studies and noticing higher HPV prevalence in this study compared to the patients with cancer, will give credence to the fact that there is spontaneous resolution of HPV infection irrespective of the oncogenic potential of the virus while the remaining persistent strains cause the dysplastic changes cumulating to cervical cancer [4, 5]. Mixed infection among women has been documented [2] and multiple infections occur more in women with HIV infection and other immunosuppressive conditions [14]. A mixed infection rate of 12.5% was observed involving strains 18 and 35, which is lower than the occurrence of multiple infections in 33.5% of the women with HPV in the Ibadan study [14]. The retroviral status of the participants in this study was, however not tested. The peak of prevalence of HPV in this study was noticed in 2 age groups of 20 and 29 years and 30 and 39 years of age. These groups represent the women in their reproductive peak who are sexually active and thus not surprising so that the high oncogenic strains are more in the age distribution. This shows an indirect link between sexual activity and human papilloma virus infection as noted in other studies. Similar findings of preponderance of HPV infection in women less than 25 years in Mexico was also noted, though a peak at 55-64 years was observed [15]. This may indicate possible variations in sexual practices or other cofactors that may reduce the immunity of the body to handle already existing virus enabling expression of the human papilloma virus.

It was observed that patients with higher parity (>3) had about two times higher risk of human papilloma virus infection. HPV. Similar observations were noted by Fadahunsi et al in Nigeria [15]. Various reasons have been put forward to explain the preponderance of positivity among this population such as hormonal changes in pregnancy resulting in reducing immunity, to the exposure of the ectocervix during repeated child birth resulting in easy attachment of the human papilloma virus in addition to damage of the cervical epithelium during childbirth and easy accessibility of the virus to be incorporated into the cellular matrix of the cervix [16]. A comparison of women's educational status to the occurrence of hrHPV showed a statistically significant relationship between no education and the presence of the presence of oncogenic strains of the virus. Lack of education had been associated with the high risk sexual practices and a poor health seeking attitude [17, 18]; cumulating in the increased presence of sexually transmissible infections like HPV.

The influence of the sexual orientation of women as a contributory factor to the occurrence of hrHPV is evident in this study as majority of the women with more than one lifetime partner and those with spouses with multiple sexual partnersare associated with higher risk of acquiring oncogenic strains of HPV. The lack of statistical significance may be linked to the population size of affected persons relative to the general sample size, nevertheless the positive association between these factors is evident by the OR ratios analyzed. This role of multiple sexual partners in acquiring HPV infection was also observed in a study done in Columbia [19]. The presence of multiple sexual partners inadvertently increases the risk of acquisition of sexually transmissible infections. This fact was also collaborated in this study which showed that a significant number of patients with previous STIs having highly oncogenic strains of HPV. Jensen et al had established a link between heavy smoking and persistence of oncogenic strains of HPV [20]. These had been attributed to the nicotinic inhibition of the phagocytic property of cervical macrophages resulting in the persistence of the virus. Contrary to the above, this study did not elicit any association between smoking and the occurrence of HPV. Cultural inhibitions in the environment under study frown at smoking among women, which may have contributed to the low number of smokers in the study. Nevertheless, smoking was found not to be associated with hrHPV, hence might be a confounder in the expression of HPV in previous studies.

This study is limited by the fact that it is hospital based; hence community based-study is strongly advised to increase the power of the study. The association of human immunodeficiency virus infection with higher incidence of oncogenic strains of human papilloma virus had been demonstrated by various studies but that was not taken into consideration in this study, hence a survey on the effect of human immunodeficiency virus infection in this environment is advocated in subsequent reviews.

## Conclusion

The high prevalence of the high risk HPV genotypes noted in this study exposes the magnitude of the burden of HPV infection in our environment. There is need to increase the level of surveillance on females at risk of cervical cancer in this environment, since significant proportion of highly oncogenic strains with a high propensity to transformation to malignancy were observed in this study. Furthermore, there is need to encourage Government agencies to promote a more inclusive HPV vaccine such as Gardasil-9 in their national immunization scheme which has a wider coverage as against those currently available in most countries. This should be administered to girls at the appropriate age if transmission of HPV is to be reduced. There is also need for sexual behaviour modification in order to reduce the impact of the risks factors identified in this study.

### What is known about this topic

- Oncogenic strains of HPV are responsible for 99% of cervical cancer
- Natural history of cervical cancer
- HPV genotyping is a screening method for cervical cancer

### What this study adds

- This is the first study of its kind in South-South Nigeria
- Revealed a relatively high prevalence of oncogenic strains of HPV in the population studied, reinforced the sexual links in the acquisition of HPV virus and identified genotype 18 as the predominant HPV present in the women in the South-South Nigeria
- Highlighted the need for an urgent sexual reorientation to reduce prevalence of high risk HPV strains and highlighted the importance of education in reducing the prevalence of oncogenic strains of HPV
